# Caregivers' Barriers to Disclosing the HIV Diagnosis to Infected Children on Antiretroviral Therapy in a Resource-Limited District in South Africa: A Grounded Theory Study

**DOI:** 10.1155/2012/402403

**Published:** 2012-12-10

**Authors:** Sphiwe Madiba, Kebogile Mokwena

**Affiliations:** ^1^Department of Environmental and Occupational Health, School of Public Health, Faculty of Health Sciences, University of Limpopo, Medunsa Campus, P.O. Box 215, Medunsa, Pretoria 0204, South Africa; ^2^Department of Social and Behavioral Sciences, School of Public Health, Faculty of Health Sciences, University of Limpopo, Medunsa Campus, P.O. Box 215, Medunsa, Pretoria 0204, South Africa

## Abstract

We used a grounded theory approach to explore how a sample of caregivers of children on antiretroviral treatment (ART) experience HIV disclosure to their infected children. This paper explores caregivers' barriers to disclosing HIV to infected children. Caregivers of children aged 6–13 years who were receiving ART participated in four focus-group interviews. Three main themes, caregiver readiness to tell, right time to tell, and the context of disclosure, emerged. Disclosure was delayed because caregivers had to first deal with personal fears which influenced their readiness to disclose; disclosure was also delayed because caregivers did not know how to tell. Caregivers lacked disclosure skills because they had not been trained on how to tell their children about their diagnosis, on how to talk to their children about HIV, and on how to deal with a child who reacts negatively to the disclosure. Caregivers feared that the child might tell others about the diagnosis and would be discriminated and socially rejected and that children would live in fear of death and dying. Health care providers have a critical role to play in HIV disclosure to infected children, considering the caregivers' expressed desire to be trained and prepared for the disclosure.

## 1. Introduction

South Africa has made a remarkable progress in rolling out antiretroviral treatment (ART). In the past seven years the country has established the largest public-sector ART programme in the world, with approximately 919 923 people on treatment by the end of 2009 [1]. As with adults, a considerable progress has been made in providing ART to children; by the end of 2009, about 86 270 children younger than 15 years of age were on ART [2]. With the largest ART programme in the world, South Africa is experiencing significant public health benefits associated with improved treatment. The increasing availability of ART has resulted in more HIV-infected children surviving to older age and adolescence [3]. Similar to well-resourced countries [4, 5], this population of children is now older, healthier, and living with HIV as a chronic illness. 

However, the prospect of a longer lifespan for children on ART brings new challenges, and the issue of HIV disclosure becomes more significant because of the multiple benefits of disclosure for the children and their caregivers [4, 6–8]. Disclosure is related to good or improved adherence to ART medications and influences children's participation in healthcare decision-making [9–13]. It also enables children to understand HIV infection and make sense of their disease-related experiences as well as the importance of adherence [11, 14, 15]. 

Despite the growing evidence of the benefits of disclosure and the increasing population of children and adolescents on ART, HIV disclosure to infected children continues to be delayed until older childhood and beyond [16]. Recently, data from Ethiopia show that HIV disclosure rates to infected children in resource-limited settings remain low [17]. Very little is known about when, why, and how caregivers disclose HIV to infected children [18]. Caregivers may be reluctant to disclose for various reasons that were previously documented in well-resourced and resource-limited countries [5, 12]. However, Kallem and colleagues [14] argue that the low rates of disclosure to HIV-infected children are partly due to lack of guidelines on when and how to disclose HIV to infected children.While guidelines on disclosure of HIV infection among adults have received considerable attention [5], until recent guidelines by the World Health Organisation [19], there were no such guidelines for assisting caregivers and providers to make decisions about disclosing HIV to infected children in resource-limited settings [6–8, 20]. One other issue that makes disclosure most difficult for caregivers is knowing when and how to talk about HIV to their children [21, 22]. Caregivers' lack of skills on how to disclose HIV to infected children was previously reported [8, 23, 24].

Given that disclosure to HIV infected children in limited resource settings is delayed until older childhood [11, 18, 25], many children, who started ART in South Africa in the past seven years, are presumed to be at the age where they should know their HIV diagnosis. Delaying HIV disclosure to these children is likely to result in accidental disclosure because children are exposed to HIV- and AIDS-related information in their communities and schools and through the media. Accidental disclosure could lead to misinformation, confusion, bitterness, and limited opportunities for processing the information in a supportive environment [13]. However, research on what influences caregivers decision making process to disclose HIV to infected children in developing countries is limited. It is further argued that the social and cultural contexts for HIV disclosure practices of families affected by HIV in developing countries are likely to differ [26, 27]. It is imperative that health care providers and researchers in South Africa, the country with the largest paediatric ART programme in the world [1], understand disclosure of HIV to infected children to develop interventions to support caregivers in disclosure. We used a grounded theory approach to explore how a sample of caregivers of children on ART in a resource-limited district in South Africa view and experience HIV disclosure to their infected children. This paper examines caregivers' barriers to disclosing the HIV diagnosis to their children.

## 2. Material and Methods

Data described here were collected as part of doctoral grounded theory study conducted in partial fulfilment of the requirements for a doctoral degree from the School of Public Health, University of Limpopo, Medunsa Campus, South Africa. Data were collected between November 2009 and March 2010. 

### 2.1. Setting

Focus-group (FG) interviews were conducted with caregivers of HIV-infected children receiving ART in a paediatric clinic of an Academic Hospital. The clinic at the Dr George Mukhari academic hospital started with paediatric ART in 2004 and provides treatment and care to children from urban, periurban, and informal settlements in a resource-limited district of Gauteng province in South Africa. 

Caregivers were recruited as they waited for consultation and medication during the routine monthly visits for their children, and scheduled appointments were made with caregivers who met the inclusion criteria. The criteria for selection for FG interviews were being the caregiver of an HIV infected child aged 6–13 years and receiving ART. Caregiver biographic and demographic information such as their age, gender, level of education, employment status, relationship with child, and the age of the child were carefully considered to maximize variation. Caregivers were purposely selected to ensure a full representation of the age range of 6–13 years to inform the study. Caregivers of children younger than 6 years and older than 13 years were excluded. One other critical criterion was a caregiver's report that the child knew his/her HIV diagnosis, which was used to assign caregivers to either the disclosed or the nondisclosed groups. For the purpose of this study, we defined a caregiver as the biological mother, biological father, grandmother, grandfather, foster parent, or other relative who performs primary caregiving functions for the child routinely or on a daily basis.

### 2.2. Data Collection

FG interviews were conducted by the lead researcher experienced in facilitating focus groups. A trained research assistant assisted with recruitment and FG interviews. Two open-ended FG guides, one for disclosed and one for non-disclosed caregivers, were used for the FG interviews. The guides were developed in English and translated into Setswana, a local language spoken by most caregivers in the study site. FG interviews were conducted in Setswana and were audio recorded with the participants' permission and lasted for about 60 to 90 minutes. A written informed consent was obtained from individual caregivers prior to the start of the FG interview. The caregivers received R50.00 (equivalent US $8) to cover their transport cost, and refreshments were provided at the end of the FG interviews. A total of nine FG interviews were conducted with disclosed and non-disclosed caregivers of children on ART. One FG interview was conducted with a mixed group of disclosed and non-disclosed caregivers, three with non-disclosed and disclosed caregivers, respectively. Each FG interview had an average of seven participants with a total of 52 caregivers. Two follow-up FG interviews were conducted with 12 caregivers. 

The sociodemographic information of the caregivers and the children in their care was collected at the end of the FG interviews using a brief self-administered questionnaire. 

The Medunsa Research Ethics Committee of the University of Limpopo granted ethical approval for the study, and a permission to conduct the study was obtained from the hospital management of Dr George Mukhari Academic Hospital. Participation in the study was voluntary, and the researchers ensured confidentiality throughout data collection. Informed consent was obtained from participants prior to the FG interviews.

### 2.3. Data Analysis

The constant comparative data analysis as described by Strauss and Corbin [28] guided the analysis of the data for this study. FG interviews were transcribed verbatim in Setswana by a transcriptionist and translated to English by the lead researcher. Each transcribed FG interview was reviewed for accuracy by replaying each interview recorder whilst reading and translating the transcript. Transcripts were imported into NVivo version 8, a computer software package for qualitative analysis, which was used for coding all the FG interviews. The primary coding of the transcripts was undertaken by the lead researcher. Analysis began with the development of a code list from multiple readings of a number of transcripts. The code list was reviewed by the lead researcher and the co-author for consensus on the definitions of the themes and subthemes. This was followed by open coding, to identify major themes and categories that emerged from the data. The lead researcher and the coauthor met frequently to review themes, and new emergent themes were integrated in the coding process. Axial coding was used to draw tentative connections and relationships between the codes and categories that were generated through open coding [28]. The simultaneous processes of data collection and analysis permitted ongoing verification of codes and categories. Data triangulation, transcribing verbatim, peer debriefing, and analyzing data using computer software were employed to attain trustworthiness. 

Because this paper is focused on caregivers' barriers to disclosing the HIV diagnosis to their children, we have confined this analysis to FG interviews with non-disclosed caregivers. Responses provided on the caregiver and child demographic data were analysed using Stata version 10.0 [29].

## 3. Findings

### 3.1. Sample Description

Demographic characteristics of caregivers are presented in Table 1. A total of 25 caregivers of children on ART participated in three FG interviews and one follow-up FG. They ranged in age from 20 to 70 years. Half 13 (52%) of the caregivers were the biological mothers of the children. The caregiver employment status revealed that more than half 14 (56%) were unemployed and that the majority 22 (88%) depended on child support grant. A quarter 6 (24%) of the caregivers were single while more two thirds 15 (60%) were married. With regard to their educational attainment, two thirds 15 (60%) had a high school and tertiary education and a quarter 8 (28%) had a primary education. More than half 14 (56%) were HIV positive and more than a third 9 (36.4) did not know their HIV status. 

Demographic information on children was collected from the caregivers and is presented in Table 2. The children ranged in age from 6 to 13 years. Almost all (96%) of the children were in primary school. More than two thirds 14 (66%) were diagnosed between 6 and 12 years with 8 (32%) diagnosed between 11 and 12 years. The mean age of diagnosis was 6.8 years. All of the children were on ART and the mean duration on ART was 3.2 years. Ten (40%) children were maternal orphans. 

### 3.2. Themes

Analysis provided significant findings regarding caregivers' barriers to disclose the HIV diagnosis to infected children. Three main themes, caregiver readiness to tell, right time to tell, and the context of disclosure, emerged from the data. A summary of themes and subthemes is presented in Table 3. 

#### 3.2.1. Caregiver Readiness to Tell

Caregiver readiness to tell is the perceived preparedness of the caregiver to communicate the diagnosis and HIV-related information to the child. Caregiver readiness to tell characterized all of the decisions to disclose the HIV diagnosis to children and its lack was one of the key reasons why disclosure was often delayed.

*I do not know if I will be ready…, because I still need some answers for myself and I do not know where I can find those answers. So how can I tell the child, I even wish that somebody can tell the child on my behalf (37 year-old HIV-positive biological mother of a 6 year-old girl).*

Throughout the focus groups, caregivers mentioned that they were not ready to tell the child about their HIV diagnosis. This is how caregivers responded to a question as to what they meant by being ready.

*Readiness is about me and the child…, is not it? I will be ready and for the child it will be time for her to know. The child will be ready and able to ask me questions…, like “mama why is it like this…, what happened”…, and I will be able to answer her correctly and she will also be able to understand well (38 year-old HIV-positive biological mother of a 9 year-old girl).*

The caregivers were also asked to explain what should happen to make them ready to tell. This is how caregivers describe their understanding of the process of getting ready to tell.

*I do not have the courage yet…, I think maybe I can appreciate professional help to be advised what to do…, thereafter I think I will be able to tell the child (38 year-old HIV-positive biological mother of a 9 year-old boy).*

#### 3.2.2. Knowing How to Tell

Disclosing HIV to children depends on the caregiver self-readiness. Caregivers reported inadequacy in dealing with disclosure and conceptualized this as not knowing how to tell. Knowing how to tell influenced the caregivers' decision to disclose and emerged as one of the key reasons for delaying disclosure to children.

*I want her to know…, but I do not know how to approach her…, I am not sure how to approach her (32 year-old aunt of a 10 year-old girl). *

#### 3.2.3. Knowing What to Tell

Caregivers were also fearful that children might ask questions that they would have difficulty in responding to and conceptualized this phenomenon as not knowing what to tell. Caregivers felt that they did not have adequate HIV-related information to explain the diagnosis to children.

*Eish!!! I am also afraid…, I am afraid of her questions…, I do not know how to talk…, and I do not know how to answer her. She will question me asking me when, why…, it's difficult (30 year-old HIV-positive biological mother of an 8 year-old girl).*

*I do not know how to approach her, do I say “my girl you are HIV positive” I do not know where and how to begin the conversation (37 year-old HIV-positive biological mother of a 6 year-old girl).*

Caregivers believed that knowing how to tell entailed finding ways or approaches to disclose the diagnosis to their children.

*The way I see it…, you must find a way of knowing how to approach the child, and if he ask questions, you should answer freely without fear, and without being harsh. You need to tell him and talk to him nicely. You need to answer…, answer in the way he is asking you (38 year-old HIV-negative foster mother of an 11 year-old boy).*

#### 3.2.4. Dealing with Personal Fears

Caregivers had to deal with a number of personal fears which influenced their readiness to tell their children about their diagnosis. Caregivers anticipated that children would ask how they got infected.

*He will definitely ask how we got the disease (he and me). It will be difficult for me to tell him who infected who. But what I know is that it's his father but I won't tell him because he will hate his father. It's better for him to accept that he is sick without asking for details (26 year-old HIV-positive biological mother of a 9 year-old boy).*

*I am afraid of the questions that the child can ask…, for the child to ask me where he got it from…, what happened? (30 year-old HIV-positive biological mother of an 8 year-old girl).*

In addition, biological caregivers had to deal with the strong feelings of self-blame, self-condemnation, and guilt they had for infecting their children with HIV.

*I was afraid that the child will reject me…, and these children are blaming us now, they are blaming us…, they have anger (30 year-old HIV-positive biological mother of a 12 year-old girl).*

Most of the caregivers were also fearful about explaining the nature of the illness to the children. Caregivers were convinced that the children would ask how they got the disease. For caregivers, explaining the nature of the illness also meant explaining how the child got infected and consequently discusses the caregivers' sexual activities.

*I have fears that I must tell the child how HIV and AIDS is transmitted you see…, it is difficult to tell the child about the role of sex in the transmission of HIV and AIDs. That is why I am saying the child must grow up a bit and be able to understand…, when he understand you can tell him what happens, that when you have sex with someone who is HIV positive you get the virus and if you are pregnant and you are HIV positive the child can also get infected (45 year-old HIV-positive biological mother of an 11 year-old boy).*

#### 3.2.5. The Reaction of Others to the Disclosure

Caregivers were fearful that if the diagnosis was revealed the child might not be able to keep it a secret but would tell other children at school and in the neighbourhood that he/she has HIV. The failure of the child to keep the secret would result in the child being exposed to discrimination, mockery, and social rejection.

*I am afraid that he will be discriminated against at school…, you will find that other children will refuse to play with him and say that he is HIV positive. At home in the family, other children will not want to play with him…, they will say he is HIV positive. I haven't told him that he is HIV positive so that he can be accommodated at home and at school (45 year-old HIV-positive biological mother of an 11 year-old boy).*

#### 3.2.6. Support for Disclosure

Caregivers delayed disclosure because they felt inadequate in dealing with disclosure, and most would welcome the support of healthcare providers when they were due to disclose the diagnosis to their children.

*I was thinking that when the time comes for telling the child, there should be someone with me…, like a doctor (47 year-old foster mother of an 8 year-old boy). *

Though caregivers would welcome the support of healthcare providers to disclose to their children, the support was seen as taking the form of empowering the caregivers to make the disclosure.

*My problem is that I am not trained to sit down with the child and tell him that he is HIV positive. I am not sure how to handle him if he reacts negatively. So at least if there was a professional, a nurse or whoever who can prepare us, who can teach us that when you want to tell the child that he is HIV positive you need to take the following steps. At least then can we have the power to say this is how I will tell the child and if he asks this I will answer this way (45 year-old HIV-positive biological mother of an 11 year-old boy).*

#### 3.2.7. The Right Time to Tell

Caregivers delayed disclosure because they believed that it was not the right time to tell the child. The right time for the child to be told was described as the time when the child is perceived to have the ability to understand the HIV diagnosis.

*I am afraid of telling him…, I think that maybe I should wait…, maybe until 18 years when he is much older I will tell him. Now he is 11 years, he does not know what is right or wrong he is just playful. That is why I do not feel free to tell him that he is HIV positive (36 year-old HIV-positive biological mother of an 11 year-old boy).*

The caregivers responded to a question as to what they perceived as the right time to tell the child about the HIV diagnosis.

*You cannot tell a 3 year or 4 year old that he is HIV positive…, he does not understand what that is, so…, I think the right time is when you see that the child understands that there is a disease called HIV, and that when you explain he will understand. Even if you explain, a 3 year old will not understand what you are talking about. So I think there is a right time…, the time when you see that it's time for me to tell the child because he will understand (38 year-old HIV-positive biological mother of a 9 year-old girl).*

#### 3.2.8. Understanding the HIV Diagnosis

Understanding the HIV diagnosis was one of the reasons some caregivers delayed disclosure to children. Caregivers delayed disclosure because they perceived the child as not being old enough or mature to understand the diagnosis.

*I am waiting for the child to grow up a bit so that he can understand everything, and then I will tell him. So…, I think that when I tell him…, when he gets older and I tell him he will be able to capture everything quickly and understand what is happening. But for now I thought that he is still young and would not understand…, so…, he will be able to understand at that time when I tell him. So…, I want to wait until he is 10 years then maybe his understanding will be alright to capture everything that I tell him (46 year-old foster parent of a 7 year-old boy). *

#### 3.2.9. Developmental Issues

Caregivers also delayed disclosure till puberty and view telling children about their diagnosis during puberty as a means of protecting others from HIV infection.

*I think it is right that the child know about her status because she is growing and she will reach the age when she must go out with boys, and these days children mature early. If she knows, maybe she will play safe. I think when she reaches adolescence she will know what to do and what not to do (32 year-old aunt of a 10 year-old girl).*

#### 3.2.10. The Child's Reaction to the Diagnosis

Throughout the disclosure process the caregivers were fearful of the children's reaction to the disclosure of their diagnosis, and most were of the opinion that the children would be hurt by the disclosure.

*I am afraid that this thing will affect her…, and she will grow up with this thing…, and she will talk about it even if it is not necessary to be talking freely (30 year-old HIV-positive non- biological mother of an 8 year-old girl).*

*I am afraid of telling him and I am also afraid of how he will react when I tell him. He is very cheeky and gets easily angry. I do not know how he is going to react when I tell him (26 year-old HIV-positive biological mother of a 9 year-old boy).*

#### 3.2.11. Death and Dying

Thoughts of death and dying characterized the discussion of the disclosure of the HIV diagnosis to children in this study setting. HIV and AIDS have been contextualised as meaning death and dying by caregivers. This thought was expressed by caregivers who were afraid that if the children knew about the diagnosis they would think that they were going to die.

*The thing is…, our children…, whenever you mention HIV…, they know that AIDS kills…, so when you tell her, she knows that she is going to die. That is what we are afraid of…, because the child will live knowing that she is going to die. Is not it we hear that AIDS kills…, that when you have AIDS you die? When you tell the child about it she will think she is dying (37 year-old HIV-positive biological mother of a 6 year-old girl).*

The children in this study have been exposed to reports of the devastating effects of HIV and AIDS at home and through the media. Some of the caregivers believed that this exposure resulted in the children having negative perceptions of HIV. Caregivers delayed telling their children about the diagnosis for fear that they might thereafter live in constant fear of death and dying.

*The child must know about his HIV status. It's just that we parents sometimes feel that if you tell the child he will be hurt because on TV, they see all the stages that a HIV positive person goes through, from the beginning to the end, they see how a HIV positive person ends. So…, you think that if you tell him, because he saw the picture of a HIV positive person, he will think that he will also end like that. So this is the fear that we have…, that if I tell him he will live in fear (38 year-old HIV-negative foster mother of an 11 year-old boy).*

Some of the children had seen their parents or other members of the family die, and the caregivers were afraid to disclose because they believed that when children learnt that they had the same disease that killed their parents they would live in fear of death and dying. The death of the biological parents influenced HIV disclosure to children.

*So…, she also ask about her mother…, she wanted to know how her mother died…, what she died from…, then I was scared to tell her…, I think that if I tell her, she will be hurt and will think that she will also die (57 year-old grandmother of an 8 year-old girl).*

*I am afraid to tell him because it will hurt him…, he will think that he is also going to die (46 year-old HIV-positive biological father of a 10 year-old boy).*

#### 3.2.12. A Long History of Illness

Disclosure in this study was further characterized by the history of long illness of the children before diagnosis and the initiation of ART. Some caregivers delayed disclosing the HIV diagnosis because of the previous severity of the child's illness.

*It is difficult for me because my child suffered from many illnesses including a stroke and epilepsy. I cannot tell him (46 year-old HIV-positive biological father of a 10 year-old boy).*

*I think the time will come when I will be able to tell him myself, for now he is still sickly but he will be okay and I do not think I can tell him now (46 year-old HIV positive biological father of a 10 year-old boy).*

## 4. Discussion

This paper describes caregivers' barriers to disclose HIV to infected children receiving ART. We found that caregiver readiness to tell influenced HIV disclosure to children in this study. Disclosure was delayed when the caregivers believed that they are not ready to tell the infected child about their HIV diagnosis. The process of the caregivers determining whether or not they are ready to disclose to the infected child is characterized by their struggle to weigh their own perceptions of readiness to tell against the children's readiness to know. Similarly, Ledlie [30] found that prior to disclosure, caregivers reported that they have to be ready first to tell their infected children the name of their illness. Our findings are also in line with Dematteo and colleagues [31] who argue that, for disclosure of the child's HIV diagnosis to occur, adults have first to trust in their own readiness and competency to disclose. The data show that the HIV-positive status of the caregiver greatly influenced caregiver readiness to tell. More than half (56%) of the caregivers consisted of HIV-positive biological caregivers of infected children. Data from several studies show that caregivers who disclose early tend to be caregivers who are not biological caregivers of the infected children [12, 13, 30, 32–34]. Disclosure is especially difficult for HIV-infected biological caregivers who may feel responsible and guilty for infecting the child and fear that children may blame them [13, 15, 30, 33, 35]. Furthermore, disclosure is difficult for HIV-infected biological caregivers who might be particularly worried about their children learning of their illness given the stigma associated with the disease [17].

Disclosure was also delayed because caregivers had to first deal with personal fears which influenced their readiness to disclose to their infected children. Fundamental to the personal fears of caregivers was their anticipation that the children would ask how they got infected. Answering that question was perceived as problematic, particularly in the view of caregivers' perception that HIV is a sexually transmitted disease. Answering that question would compel biological caregivers to give a detailed explanation of their sexual activities in an attempt to explain the nature of the illness to their infected children. Explanations about how the child got infected may be difficult for biological caregivers to put into words [32]. Addressing the subject about the sexual activities related to the transmission of HIV was the most difficult thing for caregivers to do, especially in a context where the children were presumed to be perinatally infected. As mentioned, biological caregivers had to deal with feelings of guilt for not protecting their children from the HIV infection, suggesting that such feelings of guilt may result in biological caregivers delaying HIV disclosure to their children. The data concur with previous findings [30, 32]. 

Disclosure was also delayed because caregivers felt unskilled in dealing with disclosure to infected children and conceptualized this as not knowing how to tell. In order to know how to tell, caregivers reported that they needed to have a strategy or an approach to aid in telling the infected child about their diagnosis. This finding is in line with current and previous studies [5, 7, 24, 36, 37]. Most caregivers registered a number of concerns about their ability to disclose to infected children, namely, that they had not been trained in how to tell their children about their HIV diagnosis, that they did not know when and how to begin the disclosure conversation, that they had not been taught how to talk to their children about HIV, and that they did not know how to deal with a child who would react negatively to the disclosure. Caregivers in this study and in other studies desired to be trained and supported by healthcare providers for disclosure [7, 18, 20, 36, 38]. Data show that when caregivers are trained, they disclose by adapting the information to the infected child, taking into consideration the child's age or developmental stages [39]. 

Caregivers were also fearful of the reaction of others within their social network to the HIV diagnosis of the child. They delayed disclosure to infected children because they had fears that if the diagnosis were disclosed the child might not be able to keep a secret. If children tell others about their diagnosis they would be socially rejected, mocked, teased, and isolated by their friends at school and in the community. Protecting the child from HIV-related stigma and discrimination was one of the key reasons caregivers delayed disclosing the HIV diagnosis to children in this study. The heightened fear of discrimination in this study is mainly as a result of biological caregivers' past experiences. Caregivers who experienced personal discrimination when they disclosed their own HIV status worried that the same would happen to their infected children. Similarly, Domek [23] argues that the anticipated negative consequences related to stigma are very real for most caregivers and may delay or prevent HIV disclosure to infected children. Caregivers, in this study and others, were of the opinion that when children are too young, they are not capable of keeping secrets, subsequently delayed disclosure till older age and adolescence [7, 11, 14]. 

The right time to tell was often used to explain caregiver readiness to tell; caregivers often delayed disclosure to infected children because they believed that it was not the right time to disclose. Time in this context refers to the child's readiness to be told about their HIV diagnosis and may or may not be associated with the age of the child. The right age to tell is contextualised as the time when the child is perceived to have the ability to understand the diagnosis and is influenced by the child's intensity of asking questions about the diagnosis, the child's developmental issues, and the possible reaction of the child to the disclosure. Similar conditions were found to influence disclosure to infected children in previous studies [30, 36, 37, 39]. Disclosure was also influenced by the anticipation of a negative reaction from children upon learning of their HIV diagnosis. Caregivers felt that the children would be sad, would be hurt, would lose hope, and would live in fear of dying. Similar findings were previously reported by several authors in developing and well-developed settings. Although the right time to tell epitomise child readiness, often the age of the child was the reason caregivers delayed disclosure in this study.

In this study, HIV and AIDS are contextualized by caregivers as meaning death and dying, and thoughts of death and dying characterized HIV disclosure to infected children. Considering that children have been exposed to the devastating effects of HIV and AIDS, caregivers delayed disclosure because they thought that when the children learnt that they have the same disease that killed their parents they would live in fear of death and dying. Similarly, Lester and colleagues [40] reported that some of the caregivers in their study viewed telling HIV-infected children about the diagnosis as a death sentence. Furthermore, because some of the children were ill for a long time prior to diagnosis and initiation of ART, caregivers delayed disclosure to these children because they believed that the children would think that they are going to die. Thoughts of death and dying influenced disclosure to infected children even after children had been on ART and were asymptomatic. The average duration on ART for children in this study was three years. Mahloko and Madiba [34] found that duration on ART was not statistically significant with HIV disclosure, but that older child age was a determining factor for HIV disclosure to infected children. Several studies show that HIV disclosure rates to the increasing population of children and adolescents on ART remain low and continue to be delayed until older childhood and beyond [16, 17].

The data from this study suggest that caregivers attributed most of their fears for disclosure to child-related factors. This is in contrast with the findings of Mellins and colleagues [13], who argue that nondisclosure to infected children is more often the result of caregivers' own concerns. In a view supported by Dematteo and colleagues [31] for most adults, the process of HIV disclosure to infected children begins with months or years, spent in trying to accommodate or accept the child's HIV diagnosis into the adults lives. The current study supports this argument and suggests that caregivers' personal fears and concerns were among the primary reasons for delaying disclosure to their infected children. Other studies have suggested that disclosing a child's infection may equate disclosing the parents' own infection, hence the reluctance of HIV-positive caregivers to disclose the diagnosis to their infected children [13, 18, 30, 32]. 

The data presented here was collected through focus-group discussion and is subject to potential recall bias. Caregivers might not recall some of the events in the child's life like the age when the child started with ART and the age when the child was diagnosed with HIV. One other limitation is that the study could only report on barriers to disclose at one point in time and could not document how caregivers' readiness to tell changed over time and what influenced those changes. Prospective studies on disclosure to children would provide insight on how caregivers disclose over time. 

## 5. Conclusion

Various children-related reasons were cited by the caregivers for delaying HIV disclosure to infected children. However, the greatest desire for caregivers in this study was to protect their children from discrimination, social rejection, and pain. Discrimination and social rejection, clouded the disclosure process and the fear of discrimination resulted in delaying disclosure to children. Though the data suggest that caregivers delay HIV disclosure in an attempt to protect their infected children, in reality, HIV disclosure to children is delayed to a greater extent to protect the caregivers from their personal fears. Caregiver readiness to tell epitomises disclosure of HIV diagnosis to children in this study, and all of the other fears and barriers around disclosure to children are centred on their readiness. Furthermore, caregiver readiness occurs within the context of the stigma and discrimination related to HIV. It is important to point out that death and dying are also embedded in the context of caregiver readiness to disclose in this study, even after children had been on ART and were asymptomatic. 

Healthcare providers have a critical role to play in HIV disclosure to infected children, considering the caregiver's expressed desire to be trained and prepared for the disclosure. To actualise the involvement of healthcare providers in HIV disclosure to children, it is imperative that they are trained in disclosure counselling. This entails the development of context-based disclosure guidelines or adaptation of the recently released WHO guidelines to the local context of the caregivers. Such guidelines should provide healthcare providers as well as caregivers with knowledge and skills concerning HIV disclosure to children.

## Figures and Tables

**Table 1:** Demographic information of caregivers (*n* = 25).

Characteristics of caregivers	Frequency	Percent
Age		
20–30	5	19.2
31–40	10	38.5
41–50	5	19.2
51–60	4	15.4
61–70	1	3.9
Gender		
Male	2	8.0
Female	23	92.0
Marital status		
Single	6	24.0
Married	15	60.0
Widowed	3	12.0
Divorced	1	4.0
Employment status		
Employed	5	20.0
Unemployed	14	56.0
Part-time employment	2	8.0
Pensioner	1	4.0
Schooling	2	8.0
Self-employed	1	4.0
Highest level of education		
Primary	7	28.0
Secondary school	3	12.0
Completed secondary	10	40.0
Tertiary education	5	20.0
Relationship to child		
Mother	13	52.0
Father	2	8.0
Grandmother	4	16.0
Other relative	3	12.0
Foster parent	3	12.0
Caregiver HIV Status		
Negative	2	8.0
Positive	14	56.0
Unknown	9	36.4
Receiving child support grant		
No	3	12.0
Yes	22	88.0

**Table 2:** Demographic characteristics of children (*n* = 25).

Characteristics of caregivers	Frequency	Percent
Child age		
6–9 years	17	68.0
10 years	4	16.0
11 years	3	12.0
13 years	1	4.0
Child gender		
Boy	15	60.0
Girl	10	40,0
Diagnosis age		
1–5 years	11	44.0
6–8 years	6	24.0
11 years	1	4.0
12 years	7	28.0
School grade		
Primary	24	96.0
Secondary	1	4.0
Mother alive		
Alive	15	60.0
Deceased	10	40.0
Mean duration on ART	3.2	range (1–5 years)

**Table 3:** Summary of identified themes.

Caregiver readiness to tell
(i) Knowing how to tell
(ii) Knowing what to tell
(iii) Dealing with personal fears
(iv) The reaction of others to the disclosure
(v) Receiving support for disclosure
Right time to tell
(i) Right age to tell
(ii) Understanding the HIV diagnosis
(iii) Child's reaction to the diagnosis
(iv) Developmental issues
The context of disclosure
(i) History of long illness
(ii) Fear of death and dying
